# The effect of probiotics on functional constipation in adults: Double-blind, randomized, placebo-controlled study

**DOI:** 10.1097/MD.0000000000024938

**Published:** 2021-03-12

**Authors:** Fabiana Cristina Rosa Mitelmão, Cristiane de Cassia Bergamaschi, Marli Gerenutti, Karin Hächel, Marcus Tolentino Silva, Victor M. Balcão, Marta Maria Duarte Carvalho Vila

**Affiliations:** a University of Sorocaba, PhageLab - Laboratory of Biofilms and Bacteriophages; b Pontifical Catholic University of São Paulo; c Clinic of Gastroenterology Dr. Karin Häckel, Sorocaba, Brazil; d Department of Biology and CESAM, University of Aveiro, Campus Universitario de Santiago, P-3810-193 Aveiro, Portugal.

**Keywords:** *Bifidobacterium* sp., intestinal constipation, *Lactobacillus* sp., probiotics

## Abstract

**Background:**

: Evacuation disorders are common in industrialized countries, affecting an average of 15% to 20% of the healthy adult population. Probiotic therapy can reduce functional constipation and increase both the number of weekly bowel movements and quality of stools. Based on the hypothesis that a combination of more strains may provide better results for constipation and facilitate adherence to treatment, this study will evaluate the efficacy and safety of a combination of different strains of *Lactobacillus* sp. and *Bifidobacterium* sp. in functional constipation.

**Methods:**

: A single-centre trial of adults aged 20 to 80 years with intestinal constipation will be conducted at a Gastroenterology Clinic in Sorocaba, State of São Paulo, Brazil. Participants will be allocated into 3 groups receiving:

1. mixture of 3 probiotics: [*Lactobacillus acidophilus*, *Bifidobacterium bifidum*, and *Lactobacillus rhamnosus* (3 × 10^9^ CFU)];
2. mixture of 8 probiotics [*Lactobacillus acidophilus*, *Bifidobacterium bifidum*, *Lactobacillus rhamnosus, Lactobacillus paracasei*, *Bifidobacterium longum*, *Bifidobacterium lactis*, *Lactobacillus defensis*, *Bifidobacterium animallis* (8 × 10^9^ CFU)]; or
3. placebo, for 4 weeks.

The outcomes of interest will be change in frequency of weekly bowel movements, change in stool quality according to the 4–6 Bristol scale, number of volunteer withdrawal, number of adverse events and number of serious adverse effect.

**Discussion:**

The probiotic products are expected to induce beneficial changes in the intestinal microbiota, thereby increasing intestinal frequency to over 3 times a week and improving stool quality. The results can guide patients and healthcare practitioners and help in decision-making in the treatment of functional constipation.

**Trial registration and registry name:**

ClinicalTrials.gov Identifier: NCT04437147: The Effect of Probiotics on Functional Constipation in Adults: Study protocol for Double blind, Randomized, Placebo controlled Study

**Protocol Version:**

Version 01 August 30, 2020.

## 1 Introduction

Of the gastrointestinal disorders, constipation is one of the most reported conditions in clinical practice.^[1,2]^ Constipation affects 15% to 20% of adults, of which 33% are over 60 years old, with predominance in females. The medical history of patients with constipation should be analyzed together with parameters such as fecal consistency, defecator frequency, effort when defecating, feeling of incomplete evacuation, abdominal pain and discomfort, use of laxatives, surgical history, comorbidities, lifestyle and work activity.^[3]^ Functional constipation is based on symptoms of nonorganic origin and diagnosed by the diagnostic criteria of Rome IV.^[4]^ The Bristol stool scale can help patients assess and describe aspects of their stool, facilitating the recognition of constipation severity.^[5]^ Please see Annex A in supplemental digital content.

Intestinal constipation impacts quality of life and can lead to significant costs in the search for treatments and purchase of laxatives.^[1,6]^ The treatment of constipation is a challenge in that are used osmotic, stimulating, irritating, and prokinetic laxatives.^[2]^ However, it appears that up to 47% of patients are not completely satisfied due to inconsistent response to laxatives and concerns about their safety, adverse effects, taste, inconvenience, and cost.^[7]^

Currently, it is known that there is an important interaction between microbes and intestinal physiology. Therefore, probiotics have been used to treat many intestinal problems,^[8]^ such as infectious diarrhea, diarrhea associated with antibiotics, diarrhea associated with *Clostridium difficile*, hepatic encephalopathy, ulcerative colitis, irritable bowel syndrome, functional gastrointestinal disorders, necrotizing enterocolitis, and functional constipation.^[9–11]^

Although the human microbiome comprises more than 400 bacterial species, evidence has shown that a decrease in the population of *Bifidobacterium* and *Lactobacillus* in adults can result in intestinal constipation.^[12–14]^ Consequently, the probiotics used in humans for the treatment of constipation are more often of the species *Lactobacillus* and *Bifidobacterium*. According to Ceresola et al.^[8]^ these *Lactobacillus* and *Bifidobacterium* can shorten the migratory myoelectric complex period and accelerate small intestine transit, partly due to increased release of serotonin (5-HT) which has promotility effects.

Clinical trials evaluating different strains of *Lactobacillus* and *Bifidobacterium* in the treatment of intestinal constipation have observed promising results,^[15–19]^ as well as systematic review on the topic.^[20]^ However, there is no consensus on probiotic strains and dosages for the treatment of constipation.^[12,21]^

Strain selection is an important step in the production of a probiotic. Probiotics should have a beneficial effect on the host and remain viable throughout the product lifetime.^[22]^

This clinical trial will evaluated the efficacy and the safety of 2 different probiotic formulations compared to placebo. One of them is composed by 4 strains of *Lactobacillus* (*acidophilus, rhamnosus, paracasei, and casei*) and 4 strains of *Bifidobacterium* (*bifidum, longum, lactis, and animalis*), given these are widely used in the treatment of adults with functional constipation. The other formulation will constitute 2 strains of *Lactobacillus* (*acidophilus, rhamnosus*) and 1 strain of *Bifidobacterium* (*bifidum*). Thus, this study will be to confirm whether these quantity of strains significantly influence constipation.

## 2 Methods

It is a singlecenter, controlled, randomized, and blinded clinical trial outlined in accordance with the criteria set out by the SPIRIT statement.^[23]^

### 2.1 Study design and setting

This study is parallel and has the structure of superiority working with the hypothesis that the groups that will receive probiotics are more effective than the group that will receive the placebo (constituted of only fibers, vitamins, and minerals which is a conventional treatment for constipation).

Over a 4-week period, a singlecentre, double-blind, randomized, placebo-controlled study of 150 patients (50 patients per group) with functional constipation will be treated using probiotics containing 3 billion CFU of mixed strains probiotics containing 8 billion CFU of mixed strains or placebo.

The study will be conducted in Clinic of Gastroenterology Dr Karin Häckel, localized in Sorocaba, State of São Paulo, Brazil. Patient recruitment for this study will be carried out through a collaborative effort between the University of Sorocaba and physician Karin Häckel that attending in Clinic of Gastroenterology. From the university, a flyer will be sent out via e-mail and letters distributed around Campus or messages texted via cell phone, and social media will also be exploited to promote the study.

The recruitment will be until the date of November 30, 2020. After verbal and written explanation, individuals who agreed to participate in the study will sign the Free and Informed Term of Consent already approved by the Ethics Committee of the University of Sorocaba (Annex B, supplemental digital content).

### 2.2 Eligibility criteria

#### 2.2.1 Inclusion criteria

- Patients eligible for the trial are adults aged 20 to 80 years;
- Clinical diagnosis of functional constipation according to Rome IV. The Rome IV Consensus defines functional constipation as a dysfunction that manifests with difficult, infrequent and incomplete bowel movements. The constipation must have started 6 months previous and become more frequent in the last 3 months, including 2 or more of the following characteristic: involving <25% of bowel movements: effort, hardened resistance (Bristol scale 1–2), feeling of incomplete removal, sensation of anorectal obstruction, digital manoeuvres to facilitate the exit of fecal content, less than 3 spontaneous bowel movements/week and need for laxatives^[4]^;
- Granting of written informed consent.

#### 2.2.2 Exclusion criteria

- Presence of gastrointestinal diseases;
- Use of antibiotics or dietary supplements containing probiotics or prebiotics in the last 15 days;
- Pregnancy

### 2.3 Interventions

Participants will be instructed to take a sachet before breakfast, in the form of mix or powder in water and drink, and to store the sachets at room temperature. To improve adherence to the treatment, messages will be sent and/or phone calls made to verify that participants are following the protocol correctly and that these are working as intended. During the clinical trial, the use of laxatives will be prohibited.

The study is composed by 3 parallel arms:

#### 2.3.1 Active comparator: 3 billion CFU strains of probiotics

*Lactobacillus acidophilus* LA 02 ID 1688 1 billion CFU, *Bifidobacterium bifidum* BB 01 ID 1722 1 billion CFU, *Lactobacillus rhamnosus* LR 04 ID 1132 1 billion CFU, vitamin C (ascorbic acid) 45 mg, vitamin B1 (thiamine) 1.1 mg, vitamin B2 (riboflavin) 1.1 mg, vitamin D-3 40,000,000 IU/g (cholecalciferol) 34 mcg, magnesium hydroxide 0.3 g, calcium carbonate 0.5 g, natural vanilla flavor powder 0.03 g, Fos (fructooligosaccharides) qsp 3 g, for 30 days, once daily in the form of sachet.

Assigned interventions: Dietary supplement of fibers plus vitamins and minerals will be administered for 30 days once daily in sachets containing 3 billion CFU strains of probiotics

#### 2.3.2 Active comparator: 8 billion CFU strains of probiotics

*Lactobacillus paracasei* LPC 00 ID 1076 1 billion CFU; *Bifidobacterium longum* BL 03 ID 1152 1 billion CFU; *Bifidobacterium lactis* BS 01 ID 1195 1 billion CFU; *Lactobacillus casei* LC 03 ID 1872 1 billion CFU; *Bifidobacterium animalis* LMG 10508 1 billion CFU*; Lactobacillus acidophilus* LA 02 ID 1688 1 billion CFU, *Bifidobacterium bifidum* BB 01 ID 1722 1 billion CFU, *Lactobacillus rhamnosus* LR 04 ID 1132 1 billion CFU, vitamin C (ascorbic acid) 45 mg, vitamin B1 (thiamine) 1.1 mg, vitamin B2 (riboflavin) 1.1 mg, vitamin D-3 40,000,000 IU/g (cholecalciferol) 34 mcg, magnesium hydroxide 0.3 g, calcium carbonate 0.5 g, natural vanilla flavour powder 0.03 g, Fos (fructooligosaccharides) qsp 3 g, for 30 days, once daily in the form of sachet

Assigned interventions: Dietary supplement of fibers plus vitamins and minerals will be administered for 30 days once daily in sachets containing 8 billion CFU strains of probiotics.

#### 2.3.3 Placebo comparator: placebo

Vitamin C (ascorbic acid) 45 mg, vitamin B1 (thiamine) 1.1 mg, vitamin B2 (riboflavin) 1.1 mg, vitamin D-3 40,000,000 IU/g (cholecalciferol) 34 mcg, magnesium hydroxide 0.3 g, calcium carbonate 0.5 g, natural vanilla

Assigned interventions: Dietary supplement of fibers plus vitamins and minerals will be administered for 30 days once daily in sachets.

### 2.4 Measured outcomes

#### 2.4.1 Primary outcomes

Changes in the frequency of bowel movements and in stool quality will be noted down in a table which the patient must fill out with information about daily frequency and type of stool (on a scale of 1–7 of the Bristol scale, or whether there was no bowel movement).

Stool form will be assessed using the BSFS (Bristol Stool Form Scale), a simple tool for estimating intestinal transit time.^[5]^ The BSFS classifies stools into 7 categories, including type 1, separate hard lumps, like nuts; type 2, sausage-shaped, but lumpy; type 3, like a sausage but with cracks on the surface; type 4, like a sausage or snake, smooth and soft; type 5, soft blobs with clear-cut edges; type 6, fluffy pieces with ragged edges, a mushy stool; type 7, watery, no solid pieces.^[5]^ These types are categorized into slow transit (types 1 and 2), normal transit (types 3–5), and fast transit (types 6 and 7).

The metric of analysis will be the comparison between 0 and 30 days, considering that the number of effective bowel movements over 4 times a week is an effective value for the treatment and the quality of the stools from 3 to 5.

#### 2.4.2 Secondary outcomes

The number of patients and adverse events will be recorded. Adverse events are undesirable signs or symptoms that occur during the study and whose cause may or may not be causal related to the treatment. All adverse events considered possibly, probably or related to the test product will be noted down on the patient's form.^[13]^ The number of patients and adverse events will be recorded.

Serious adverse events are defined as events that are fatal, life-threatening, disabling or result in hospitalization or prolonged stay, or result in malformation, whether related to the test product or otherwise^[13]^

According to previous studies, probiotics are safe and any serious adverse event that could possibly, probably or be related to the test products will be considered unexpected. All unexpected serious adverse events will be reported to the physician. Any serious adverse event that may be related to the test product will immediately lead to discontinuation of the test product.^[13]^

### 2.5 Sample size and recruitment

**Figure 1:**
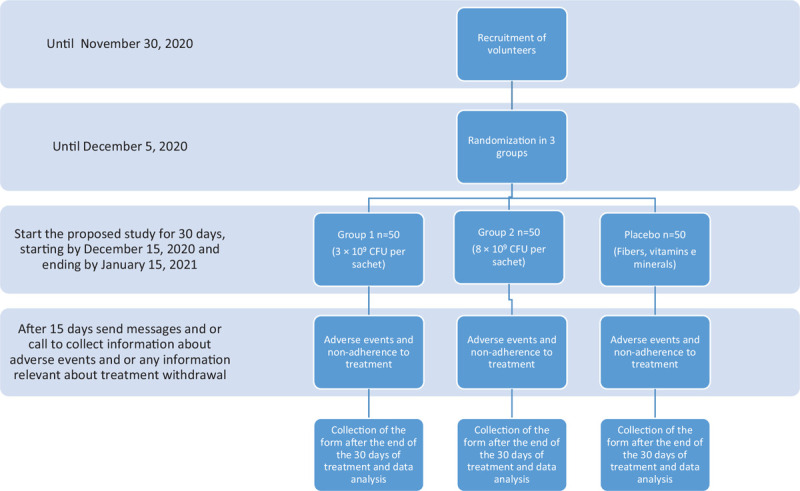
Flowchart describing study design, sample composition, and experimental protocol. BSFS = Bristol Stool Form Scale, CFU = Colony Forming Unit.

In order to achieve 80% statistical power (*P* value = .05) between the 3 treatments, 132 patients should be recruited (44 patients per group). As a dropout rate of 15% is expected that will be included in the study 152 participants. A schematic diagram of time schedule of enrolment and interventions of the research groups is summarized in Figure 1.

The strategy to achieve the appropriate registration of participants to reach the target sample size will be disseminated on social networks at the University of Sorocaba and poster at the Gastroenterology Clinic Dra Karin Hackel.

The sample size was calculated based on 2 means and their standard deviations. In preliminary studies, the correlation established using the website openepi.com.

### 2.6 Randomization and allocation concealment for treatment

After confirmation of eligibility and reading/signing of the informed consent form, patients will receive a sequential number randomized by one of researcher (FCRM). Randomization will be performed by Random Allocation Software. The patients will be stratified equally into the 3 groups in blocks of 9. Patients will receive identical sealed opaque envelopes bearing the randomized sequential number.

The patients, physician, research taste, and packaging differing only by numbering of manufacturing batches. The physician will select each volunteer according to lot number corresponding to the randomization.

Eligible patients will be allocated (1:1:1) to receive treatment, for 4 weeks, with the probiotic supplement containing 3 × 10^9^ CFU per sachet, 8 × 10^9^ CFU per sachet, or placebo. Participants of the study and all those with access to the results (outcome assessors - KH) will be blinded. All groups will receive identical sachets (same flavor, color, and packaging, with no way to differentiate one batch from another).

It will be allowed to unmask and the revealing the intervention allocated during the trial if there is a serious adverse event which the doctor will investigate if the product was really the cause.

The volunteers can withdraw from the study at any time by their own request or they can be withdrawn at any time at the discretion of the investigator for safety. Volunteers will also be withdrawn from the study in the event of treatment interruption for any reason, whether due to forgetfulness or to experiencing undue intestinal discomfort.

To promote the retention of participants and complete follow-up, a text message or call will be sent after 15 days to find out how the treatment is going, and if there is discontinuation or deviation from the intervention protocol. The following questions will be asked: Why did you not continue with the treatment? Did you have any adverse events, if any?

Volunteers will be instructed to fill out the form properly if they have any adverse reactions and what type of stool and daily frequency.

### 2.7 Data handling and record keeping

The data from the volunteers’ records will be entered in excel format and the records will be stored in a safe place in order to prove the study.

A data collection form will be used to record patient data for all participants and completed by the pharmaceutical researcher (FCRM), who will also enter the data into a database. The results will checked, in duplicate, to ensure the quality of the measured outcomes.

### 2.8 Volunteer withdrawal

The number of volunteer who dropped out of the study due to the presence of adverse events.

### 2.9 Ethical and regulatory aspects

The study protocol was approved by the Ethics Committee of the University of Sorocaba. The project is filed under the CEP-Single CAAE Number: 84003418.9.0000.5500 and was approved on 09/17/2018.

These documents state that volunteers’ informed consent is an essential precondition for participating in the clinical study. The study will begin only after approval by the local Ethics Review Board of the University of Sorocaba.

### 2.10 Data analysis planning

Preliminary data indicate that the number of evacuations weekly is 2 or 3 times higher in patients than in control volunteers. The hypothesis is that treatment will likely increase the number of weekly bowel movements from 1 to 3 times to over 4 times a week, while no changes will be seen in the placebo group.

Anova will be used in order to compare continuous variables between the intervention and control groups. Score changes between the intervention and the control groups will be compared by Kruskall–Wallis.

Analysis of adverse events (if any) and the other outcomes will be performed using the Student *t* test and Mann–Whitney *U* test. In all measured will used the intention to treat analysis. The level of significance will be at *P* < .05. The raw data will be input into the statistical software. Statistical analysis will be performed at STATA v.14.2.

All data collected on paper will be marked with a research identification number to avoid identifying the participants and stored in a locked cabinet. Only the author of the research report can access these identified data sets.

The data monitoring committee is not necessary because the statistical analysts will be blind to evaluate the outcomes data.

Volunteers will be instructed to write down any adverse events on the attached form A and if they feel the need to interrupt treatment due to inconvenience that they are unable to continue and write down what the reactions were.

### 2.11 Ethics and dissemination

The project is filed under the CEP-Single CAAE Number: 84003418.9.0000.5500 and was approved on 09/17/2018. Informed and signed consent will be obtained in the clinic participating in the research by outcome assessors (KH).

Personal information about participants will be collected and maintained in confidential before, during and after the end of trial only by researcher (KH which it will deliver to the researcher responsible for the clinical trial (FCRM).

The researchers declare no financial interests in this clinical study.

The researchers of this study declare will have access to the data set of the final essay.

The researchers will communicate the results of the trial to the participants by publishing the results in specialized journals which will be shared by text message.

## 3 Discussion

This study shall determine whether the combination of Lactobacillus and Bifidobacterium strains can improve functional intestinal constipation due to the action of bacterial flora and thereby increase the frequency of intestinal motility and improve the quality of faces.

Therefore, the combination of different strains must be evaluated, as the species may differ in their effects, as well as in aspects related to their safety.

Clinical trials are essential to assess functional therapy for constipation and identify desirable therapeutic alternatives to increase treatment effectiveness and compliance levels.

This study combining 8 strains of probiotics, as measured by benefits to the gastrointestinal system, has not yet been evaluated in a parallel group with probiotics containing 3 strains, to determine which combination will have the best action in reducing constipation, using fibers as a placebo.

A limitation of this study is that results will be based on the self-reporting of symptoms by selected volunteers with functional constipation as opposed to direct observation.

The placebo will contain no probiotic strains (only fibers, vitamins, and minerals) so that the desired effect of a significant difference in the treatment of constipation can be attributed solely to the probiotics.

## Acknowledgments

Project funding by Fundação de Amparo à Pesquisa do Estado de São Paulo (FAPESP, São Paulo, Brazil) (FAPESP Refs. No. 2016/08884-3 (Project PneumoPhageColor) and 2016/12234-4 (Project TransAppIL)), is hereby gratefully acknowledged. Funding for Victor M. Balcão through a BPE grant from FAPESP (São Paulo, Brazil) (Ref. No. 2018/05522-9, Project PsaPhageKill) is hereby gratefully acknowledged. This work also received support from CNPq, National Council for Scientific and Technological Development Brazil, in the form of Research Productivity (PQ) fellowships granted to Victor M. Balcão (Refs. No. 306113/2014-7 and 308208/2017-0). Thanks are also due to FCT/MCTES for the financial support to CESAM (UID/AMB/50017/2019), through national funds.

## Author contributions

**Conceptualization:** Marta Maria Duarte Carvalho Vila, Fabiana Cristina Rosa Mitelmão.

**Formal analysis:** Marcus Tolentino Silva.

**Investigation:** Karin Hächel.

**Writing – original draft:** Marta Maria Duarte Carvalho Vila, Fabiana Cristina Rosa Mitelmão.

**Writing – review & editing:** Cristiane de Cassia Bergamaschi, Marli Gerenutti, Victor Manuel Balcão.

## Supplementary Material

Supplemental Digital Content

## Supplementary Material

Supplemental Digital Content
