# Supplementary material for: The effect of probiotics on functional constipation in adults: Double-blind, randomized, placebo-controlled study
Source: Medicine (Baltimore). 2021 Mar 12;100(10):e24938. doi: 10.1097/MD.0000000000024938 (PMC7969262; doi:10.1097/MD.0000000000024938)
Supplement: Supplemental Digital Content [file medi-100-e24938-s001.pdf]

Stool image / Treatment days	1st DAY	2nd DAY	3rd DAY	4th DAY	5th DAY	6th DAY	7th DAY	8th DAY	9th DAY	10th DAY	11th DAY	12th DAY	13th DAY	14th DAY	15th DAY
Type 1 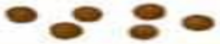 Seperate hard lumps, like nuts (hard to pass)															
Type 2 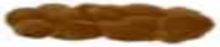 Sausage-shaped but lumpy															
Type 3 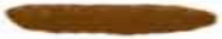 Like a sausage but with cracks on it's surface															
Type 4 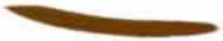 Like a sausage or snake, smooth and soft															
Type 5 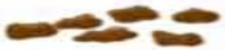 Soft blobs with clear-cut edges (passed easily)															
Type 6 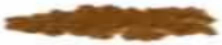 Fluffy pieces with ragged edges, a mushy stool															
Type 7 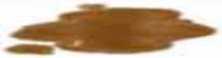 Watery, no solid pieces. Entirely liquid															
There were no evacuations															

Stool image / Treatment days	16th DAY	17th DAY	18th DAY	19th DAY	20th DAY	21st DAY	22nd DAY	23rd DAY	24th DAY	25th DAY	26th DAY	27th DAY	28th DAY	29th DAY	30th DAY
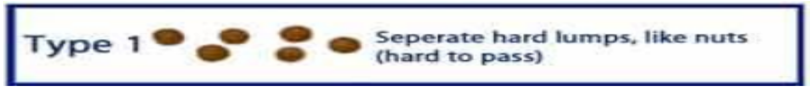															
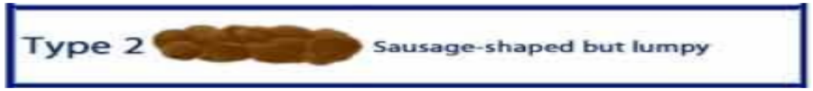															
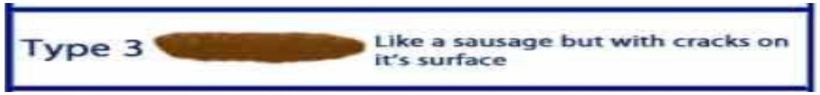															
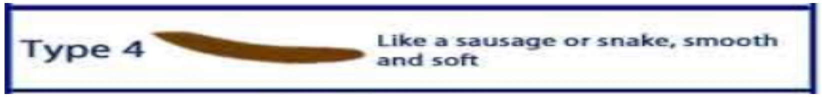															
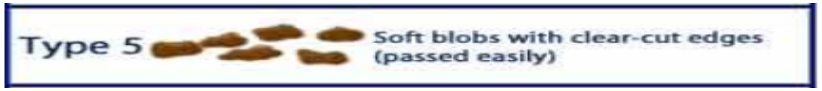															
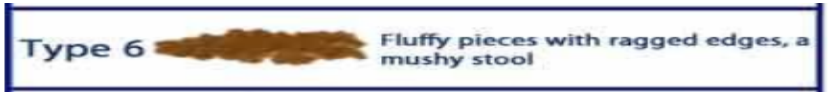															
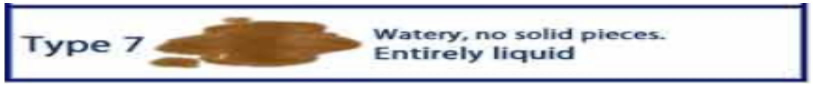															
There were no evacuations															
