# Supplementary material for: The effect of probiotics on functional constipation in adults: Double-blind, randomized, placebo-controlled study
Source: Medicine (Baltimore). 2021 Mar 12;100(10):e24938. doi: 10.1097/MD.0000000000024938 (PMC7969262; doi:10.1097/MD.0000000000024938)
Supplement: Supplemental Digital Content [file medi-100-e24938-s002.pdf]

ANNEX B

FICF - Free and Clarified Consent Term

You are being invited to participate as a volunteer in this research. Your participation is not mandatory, and at any time, you can withdraw and withdraw your consent. Your refusal will not cause any harm in your relationship with the researcher or with the Institution.

You will sign two original copies of this term, which include the telephone number and address of the researcher in charge and the research team, which may answer questions about the project and its participation. One copy will be with you and the other will be with us.

RESEARCH TITLE: The effect of probiotics on functional constipation in adults – a double-blind, randomized, placebo-controlled study.

RESPONSIBLE RESEARCHER: Profa. Dra. MARTA MARIA DUARTE CARVALHO VILA

ADDRESS: Highway Raposo Tavares Km 92.5. CEP 18023-000. Sorocaba-SP

PHONE: +55 15 2101 7029

PARTICIPATING RESEARCHER: Ms. FABIANA CRISTINA ROSA MITELMÃO

ADDRESS: RUA FRANCISCO PRADO ARO, 450, Sorocaba, SP.

PHONE: +55 15 9 8116 2387

PARTICIPATING RESEARCHER: Dr. KARIN HÄCKEL

ADDRESS: Av. Dr Armando Sales de Oliveira 371, Trujillo - Sorocaba -SP

PHONE: +55 15 3019 2100

OBJECTIVES: The general objective of this research is to compare the exposure to two formulations of probiotic mixtures, with the clinical evolution of the intestinal constipation symptom in healthy individuals.

STUDY PROCEDURES:

This is a clinical, double-blind, randomized and placebo control trial, with exposure to probiotics, for a period of 30 consecutive days, to be completed in 36 months. It aims to evaluate the efficiency of two different probiotic mixtures: (1) Lactobacillus acidophilus, Bifidobacterium bifidum and Lactobacillus ramnosus (3 billion CFU); (2) Lactobacillus acidophilus, Bifidobacterium bifidum, Lactobacillus ramnosus, Lactobacillus paracasei, Bifidobacterium longum, Bifidobacterium lactis, Lactobacillus defensis, Bifidobacterium animallis (8 billion CFU). The project is filed at CEP-Uniso under number CAAE: 84003418.9.0000.5500 being approved on 17/09/2018

RISKS, DISCOMFORT AND HOW THE PARTICIPANT WILL BE SERVED

Possible risks and / or discomforts: Diarrhea, allergy to any formulation item, nausea, vomiting. If the discomfort occurs, the participant must receive medical care at the “Gastroenterology Clinic Dra. Karin Häckel” located in the Municipality of Sorocaba, São Paulo, Brazil.

IN THIS CASE: You should inform the researcher if any discomfort occurs and if you intend to continue the research or not.

BENEFITS: Regulation of intestinal flora, increased absorption of nutrients, significant improvement in the number of weekly bowel movements and the main problems associated with bowel movements, particularly in the consistency of stools and ease of expulsion.

COST / REFUND TO PARTICIPANT: Not applicable

RESEARCH CONFIDENTIALITY: All data is for use only for the research mentioned above.

Signature of the Responsible Researcher:________________________________

RESEARCH PARTICIPANT CONSENT

I,.........., RG....................., CPF...................., declare that I have read the information contained in the Free and Informed Consent Form of the project entitled ”, which has as a responsible researcher and research team and, I was duly informed of the procedures that will be used, risks and discomforts, benefits, cost / reimbursement of participants, research confidentiality and I agree to participate.

The research participant was guaranteed:

- That all information obtained about you in this study, will be analyzed together with that of other participants, and your identification or that of other participants will not be disclosed at any time;

- That he can withdraw consent at any time, without this leading to any penalty;

- At any time, if it is of interest to you, you can have access to all information obtained about you;

- When the study is finished, you will be informed about the main results and conclusions obtained in this study.

I declare that I received an original copy of the Informed Consent Form and agree to participate in the research.

Sorocaba, .../ ..../.....................

NAME AND SIGNATURE OF THE PARTICIPANT OR RESPONSIBLE:

Full name:

Signature:
